# Reliability of Simplified Versions of Enhanced Recovery After Surgery (ERAS) in Colorectal Surgery: A Retrospective Cohort Study

**DOI:** 10.7759/cureus.103894

**Published:** 2026-02-19

**Authors:** Maria Olive, Rodrigo Moisés de Almeida Leite, Luiz Felipe B Jacomino, Francisco Tustumi, Ana Sarah Portilho, Rafael Vaz Pandini, Lucas Cata Preta Stozlemburg, Victor Edmond Seid, Lucas De Araújo Horcel, Sergio E Araujo

**Affiliations:** 1 Surgical Gastroenterology, Hospital Israelita Albert Einstein, Sao Paulo, BRA; 2 Colon and Rectal Surgery, Hospital Israelita Albert Einstein, São Paulo, BRA; 3 Gastrointestinal Surgery, Hospital das Clínicas of the Faculty of Medicine, University of São Paulo, Sao Paulo, BRA; 4 Colorectal Surgery, Hospital Israelita Albert Einstein, São Paulo, BRA; 5 Gastrointestinal Surgery, Universidade de São Paulo, São Paulo, BRA; 6 Surgical Gastroenterology, Hospital Israelita Albert Einstein, São Paulo, BRA

**Keywords:** colorectal cancer surgery, enhanced recovery after surgery, eras compliance, postoperative outcomes, predictive accuracy, simplified protocols

## Abstract

Introduction

Enhanced recovery after surgery (ERAS) programs have transformed perioperative care. However, adherence to all ERAS components can be challenging, and the contribution of individual components remains uncertain. This study evaluates ERAS compliance and compares its predictive accuracy with four simplified protocols: a) remove, ambulate, postoperative analgesia, introduce diet (RAPID), b) Basse, c) Nygren, and d) Aarts in colorectal cancer surgery.

Methods

A retrospective cohort study was conducted at Vila Santa Catarina Hospital (São Paulo, Brazil) and included patients undergoing elective colorectal resection for adenocarcinoma (2015-2022). ERAS compliance was measured by adherence to 22 components and compared with four simplified protocols (elements four to 12). Outcomes analyzed included stoma-free status, avoidance of readmission, absence of overall and severe complications, and mortality. Predictive accuracy was assessed via area under the curve (AUC) from receiver operating characteristic (ROC) curves (p<0.05).

Results

The study included 410 patients (mean age: 62.3 ±11.5 years; 53.4% female). Laparoscopic surgery was predominant (90%), followed by robotic (6.3%) and open (3.7%) approaches. Mean ERAS compliance was 76.9% (±9%). Postoperative outcomes were 66.3% stoma-free, 92.7% avoided readmission, 68.5% had no complications, 85.9% had no severe complications, and 98.1% survived. ERAS had the highest predictive value for severe complications (AUC: 0.69), similar to Nygren (0.68). Nygren outperformed ERAS for stoma-free (0.7 vs. 0.66) and complications (0.63 vs. 0.62). ERAS had low accuracy for readmission avoidance (AUC: 0.51), with RAPID and Basse performing better. No protocol showed significant superiority in predicting mortality (p>0.05).

Conclusions

Simplified protocols, especially Nygren and RAPID, showed competitive or superior predictive performance for certain outcomes. While ERAS remains a strong standard, simplified versions appear promising.

## Introduction

Enhanced recovery after surgery (ERAS) programs are composed of a series of evidence-based interventions proven to optimize the patient’s care pathway [1,2]. These programs aim to reduce perioperative complications, length of hospital stay, and improve patient recovery. Its efficiency has been demonstrated in several studies [3-6]. The ERAS Society has outlined between 16 and 25 interventions for various types of surgery [7].

However, implementing an ERAS program can be challenging. In addition to requiring an engaged multidisciplinary team of evolving surgeons, anesthesiologists, and nurses, it often conflicts with several traditional practices [8]. Furthermore, adherence to some interventions, particularly during the postoperative period, heavily depends on the effective functioning of the ERAS team [9].

High compliance with ERAS programs is essential for improving surgical outcomes and is a valuable predictor of complications [10]. However, defining a universal compliance threshold remains challenging [6]. Variability in ERAS protocols, differences in the number and characterization of interventions, and the complexities of real-world implementation hinder the establishment of an ideal benchmark [11]. Additionally, adherence to comprehensive protocols involving 16 to 25 interventions can be time-consuming and resource-intensive, raising concerns about feasibility in routine clinical practice. Despite evidence supporting ERAS principles, the relative contribution of individual components to overall patient outcomes is still unclear [12]. Some interventions may have a more substantial impact than others, while certain combinations could offer similar benefits with reduced complexity.

Therefore, refining our understanding of how adherence to simplified ERAS protocols influences patient outcomes is crucial for optimizing perioperative care. Striking a balance between protocol efficacy and practical implementation could enhance compliance and maximize benefits. This study aims to address this gap by comparing the predictive accuracy of a 22-item ERAS compliance score with four widely recognized simplified protocols, namely, a) remove, ambulate, postoperative analgesia, introduce diet (RAPID), b) Basse, c) Nygren, and d) Aarts in patients undergoing colorectal cancer surgery. Evaluating the performance of these streamlined approaches may provide insights into whether a less burdensome protocol can achieve comparable outcomes, ultimately guiding future refinements in ERAS strategies. This project was presented as an abstract at the Society for Surgery of the Alimentary Tract in May 2025.

## Materials and methods

Study settings

A retrospective cohort study was conducted at Vila Santa Catarina Hospital in São Paulo, Brazil, affiliated with Einstein hospital Israelita.

Inclusion Criteria

The inclusion criteria were adult patients aged 18 years and older who underwent elective colorectal resection for colon or rectal adenocarcinoma between January 2015 and December 2022.

Exclusion Criteria

Patients who had emergency operations, transanal conventional or minimally invasive resections, multivisceral resections, unresectable tumours, those referred to another facility during the postoperative period, and patients with cognitive or social limitations were excluded from this study.

Enhanced recovery

After surgery (ERAS) program clinical data were collected from medical records and surgical databases and prospectively inserted into the ERAS interactive audit system (EIAS), an online platform provided by the ERAS Society that allows for data storage and retrieval. It contained information on baseline demographics, perioperative interventions, complications, length of stay, mortality, and postoperative readmissions. Compliance was assessed based on adherence to 22 protocol components and compared with four simplified versions comprising four to 12 components.

Simplified protocols

The RAPID protocol [13] and Aarts et al. [14] studies include patients who underwent open and laparoscopic colorectal surgery, and each encompasses four and 11 ERAS programs’ interventions, respectively. Basse et al. [15] include patients who underwent open colonic surgery and contain seven components. Finally, Nygren et al. [16] comprise patients undergoing open colorectal surgery and 12 ERAS interventions. Each adopted program is presented in Table 1.

**Table 1:** Intervention scores ERAS interventions from each analyzed study were used to calculate the score for comparing the impact on outcomes. Some interventions are subdivided into multiple items that must all be implemented to achieve adherence. 1- ERAS: Enhanced Recovery After Surgery; 2- RAPID Protocol: Simplified 4-item bundle: Remove (nasogastric tubes/urinary catheters), Ambulate (early mobilization), Postoperative analgesia (minimal opioids), and Introduce diet (early oral nutrition); 3- Aarts Protocol: Simplified 11-item recovery program; 4- Basse Protocol: Simplified 7-item recovery program; 5- Nygren Protocol: Simplified 12-item recovery program. ERAS- enhanced recovery after surgery.

Items			ERAS^1^	RAPID^2^	Aarts^3^	Basse^4^	Nygren^5^
	Preoperative						
Preadmission Counseling			1		1		1
Preoperative Optimization			2				
Preoperative Nutritional Support			3				
Management of Anemia			4				
No Premedication			5		2	1	
Postoperative Nausea and Vomiting Prevention			6				
Antibiotic Prophylaxis			7				2
Selective Bowel Preparation			8		3		3
Reduced Fasting Duration			9		4		
	Intraoperative						
Standard Anesthetic Protocol		Multimodal Anesthesia	10.1		5	2	4
		Short-Acting Anesthetic Agents	10.2			3	
Intraoperative Fluid Therapy			11		6		
Maintenance of Normothermia			12				
Minimally Invasive Surgery			13				
Avoidance of Prophylactic Drains			14		7.1		5
Avoidance of the Nasogastric Tube			15	1.1	7.2		6
	Postoperative						
Minimal Use of Opioids			16	2		4	7
Thromboprophylaxis			17				8
Postoperative Fluid Therapy		Avoidance of Salt and Water Overload	18.1	1.2			
		Avoid an Excess of Postoperative Weight Gain	18.2				
Early Removal of the Urinary Catheter			19	1.3	8	5	
Stimulation of Gut Motility			20		9	6	9
Postoperative Nutritional Care		Nutritional Support	21.1				10
		Early Oral Nutrition	21.2	3	10	7.1	11
Early Mobilization			22	4	11	7.2	12

Follow-up and outcomes

All patients were followed for 30 days after surgery or until postoperative death, whichever occurred first. The primary outcomes were stoma-free status (absence of a stoma within 30 days after surgery), no readmission within 30 days, absence of any postoperative complications, absence of severe complications, and no postoperative mortality. Postoperative complications were systematically recorded and categorized according to the Clavien-Dindo classification [17]. Complications equal to or higher than Grade IIIa were classified as severe.

Ethical aspects

The study received ethical approval from the Institutional Review Board of Hospital Israelita Albert Einstein (CAAE#58157921.3.0000.0071). Due to its retrospective nature, a waiver of informed consent was requested and granted.

Statistical analysis

Descriptive statistics were used to summarize patient demographics and clinical characteristics. Continuous variables were reported as means with standard deviations, while categorical variables were represented as frequencies and percentages. The discriminative ability of the full ERAS protocol and each simplified protocol (RAPID, Basse, Nygren, and Aarts) was evaluated using receiver operating characteristic (ROC) curves and their corresponding areas under the curve (AUCs) with 95% confidence intervals. Because all five compliance scores were applied to the same patients, the AUCs represented correlated ROC curves. Therefore, head-to-head comparisons between protocols were performed using DeLong’s nonparametric test for paired AUCs, implemented in Stata through the roccomp command. This method compares AUCs by estimating their covariance structure and computing a chi-square test of equality. For each clinical outcome, we first compared the AUC of the full ERAS protocol against each simplified protocol and subsequently performed pairwise comparisons among simplified protocols. All tests were two-sided, and statistical significance was defined as p<0.05. For sample size estimation, we utilized the F test for correlation, and a total of N=320 patients was estimated for a significance level of 0.05 and power of 0.8 to detect a significant difference in the outcomes. Statistical analyses were performed using Stata 18.0 (Standard Edition, StataCorp LLC, College Station, TX, USA). Statistical significance was set at p<0.05. 

## Results

Inclusion and baseline characteristics

This study included 410 patients diagnosed with adenocarcinoma who underwent elective colorectal resection with perioperative care based on an ERAS program between 2015 and 2022 at Vila Santa Catarina Hospital. The mean age of the patients was 62.3 years (±11.5), and 53.4% were female. Most procedures were performed laparoscopically (90%), while robotic and open approaches accounted for 6.3% and 3.7% of cases, respectively. Most patients had an American Society of Anesthesiology (ASA) score of I or II (76.1%), reflecting a lower perioperative risk profile. The mean ERAS compliance rate was 76.9% (±9%). When the same perioperative dataset was evaluated according to the simplified ERAS-derived bundles, mean compliance rates were 50.1% (±27) for the RAPID protocol, 68.6% (±15) for the Basse protocol, 67.9% (±12) for the Nygren protocol, and 75.5% (±14) for the Aarts protocol. Table 2 presents the baseline characteristics of the patients.

**Table 2:** Patients’ demographics and outcomes SD: Standard deviation; ASA: American Society of Anesthesiologists physical status classification; ERAS: enhanced recovery after surgery.

	Variable	Mean	Frequency	SD or %
Total number of patients			410	100%
Sex	Female		219	53.4%
	Male		191	46.6%
Surgical Approach	Open		15	3.7%
	Laparoscopic		369	90%
	Robotic		26	6.3%
ASA Score	I/II		312	76.1%
	III/IV		98	23.9%
Age (in years)		62.31		±11.5
	ERASCompliance	75.9		±9
Outcomes	Free of Stoma	66.3		±50
	No Readmission	92.7		±30
	No Postoperative Complications	68.5		±50
	No Severe Complications	85.9		±30
	No Postoperative Mortality	98.1		±10

Outcomes

**Figure 1:**
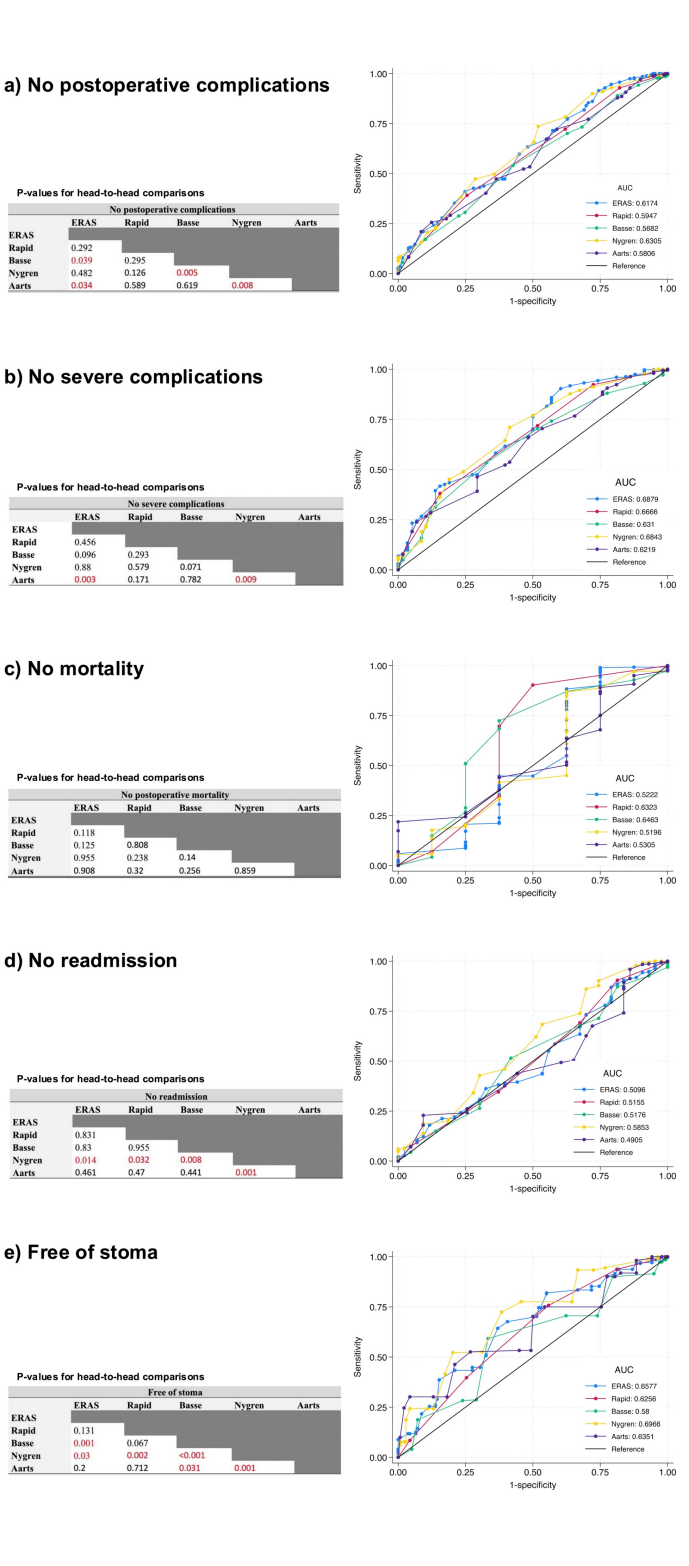
ROC curves illustrating the prediction of outcomes a) "no postoperative complications;" b) "no severe complications;" c) "no mortality;" d) "free of stoma" and e) "no readmission."  The areas under the curve (AUC) values for compliance with each protocol are displayed alongside p-values for direct comparisons between protocols. Red p-values indicate significant associations (p<0.05). Significance level was set as p<0.05. ROC curves and the consolidated tables are original data visualizations created by the authors for this specific work. ROC: receiver operating characteristic.

Predictability Figure 1 illustrates the ROC curves and the results of all head-to-head comparisons, and Table 3 summarizes each protocol’s predictive accuracy for different outcomes. It reports the AUC along with 95% confidence intervals.

**Table 3:** The area under the curve (AUC) for the ROC curves representing protocol compliance, presented with the corresponding 95% confidence intervals (95% CI) CI: confidence interval; AUC: area under the curve; LL - lower limit of the 95% CI; UL: upper limit of the 95% CI; ERAS: enhanced recovery after surgery; RAPID: remove, ambulate, postoperative analgesia, introduce diet protocol, simplified 4-item bundle - remove (nasogastric tubes/urinary catheters), ambulate (early mobilization), postoperative analgesia (minimal opioids), and introduce diet (early oral nutrition); Basse: simplified 7-item recovery program; Nygren: simplified 12-item recovery program; Aarts: simplified 11-item recovery program.

	Free of stoma	95% CI		No readmission	95% CI		No postoperative complications	95% CI		No severe complications	95% CI		No postoperative mortality	95% CI	
	AUC	LL^3^	UL^4^	AUC	LL	UL	AUC	LL	UL	AUC	LL	UL	AUC	LL	UL
ERAS	0.66	0.60	0.71	0.51	0.42	0.60	0.62	0.56	0.68	0.69	0.61	0.76	0.52	0.25	0.80
RAPID	0.63	0.57	0.68	0.52	0.42	0.61	0.60	0.54	0.65	0.67	0.59	0.74	0.63	0.35	0.91
Basse	0.58	0.52	0.64	0.59	0.42	0.61	0.57	0.51	0.63	0.63	0.56	0.70	0.65	0.40	0.89
Nygren	0.70	0.64	0.75	0.58	0.49	0.68	0.63	0.57	0.69	0.68	0.61	0.76	0.52	0.27	0.77
Aarts	0.64	0.58	0.69	0.49	0.40	0.58	0.58	0.52	0.64	0.62	0.55	0.70	0.53	0.33	0.73

Overall complications

Regarding postoperative morbidity, 58 patients (14%) experienced at least one severe complication. The most frequent events included anastomotic leak (4.6%), bowel obstruction (3%), intra-abdominal collection (1.9%), and high-output stoma (1.5%). Other recorded surgical complications were postoperative bleeding (1.2%), surgical-site infection (1.0%), fascial dehiscence (0.7%), iatrogenic bowel injury (0.7%), iatrogenic urinary tract injury (0.2%), and wound dehiscence (0.2%).

Medical complications comprised pneumonia (0.2%), acute kidney injury (0.7%), diabetic ketoacidosis (0.2%), acute pancreatitis (0.2%), cardiac arrhythmia (0.7%), thromboembolic events (0.5%), cardiogenic shock (0.5%), stroke (0.5%), acute psychiatric disorders (0.2%), and ureterolithiasis (0.2%). The Nygren protocol demonstrated the highest accuracy in predicting the absence of postoperative complications, with an AUC of 0.63. It was followed closely by ERAS, which achieved an AUC of 0.62. Both Nygren and ERAS showed significantly better predictive performance than the Basse and Aarts protocols. See Figure 1a.

The ERAS protocol demonstrated the best predictive accuracy for avoiding severe complications, with an AUC of 0.68, followed closely by the Nygren protocol (AUC: 0.68). Although all protocols had AUC values above 0.6, indicating moderate predictive capability, ERAS and Nygren were significantly superior to Aarts in this outcome. See Figure 1b.

Postoperative mortality

None of the protocols showed strong predictive power for predicting postoperative survival, and no comparison yielded statistically significant results. The ERAS protocol exhibited relatively low accuracy, with an AUC of 0.52. Meanwhile, the RAPID and Basse protocols outperformed ERAS in predicting postoperative survival, although the differences were not statistically significant. See Figure 1c.

Readmission

The ERAS protocol had low predictive accuracy for avoiding readmission, with an AUC of 0.51. In contrast, the Nygren protocol significantly outperformed all others in predicting lower readmission rates. See Figure 1d.

Free of stoma

The Nygren protocol had the highest predictive accuracy for stoma-free status, with an AUC of 0.7, followed by ERAS (0.66). ERAS and Aarts demonstrated significantly better predictive performance than Basse (p=0.001 and p=0.031, respectively). Nygren, however, was significantly superior to all other protocols in this outcome. See Figure 1e.

## Discussion

The present study investigated the predictive capability of a full 22-component ERAS program (The ERAS study program) compared with four simplified versions (RAPID, Nygren, Basse, and Aarts) [12-15] for outcomes such as stoma-free status, morbidity, readmissions, and mortality. We found that these abbreviated pathways demonstrated reasonable, albeit modest, predictive performance in the context of contemporary colorectal cancer surgery.

AUC thresholds >0.7 are traditionally used to evaluate diagnostic tests, in which accuracy at the individual-patient level is expected. Postoperative outcomes, however, are not binary states but complex, multifactorial events influenced by surgical, anesthetic, oncologic, and patient-specific factors. ERAS protocols are not diagnostic tools designed to detect disease-they are perioperative care bundles intended to reduce risk and optimize recovery at a population level. Consequently, high discriminatory AUC performance should not be expected, and the range observed in this study is consistent with the nature of the interventions being assessed.

Therefore, our intention was not to claim high precision in individual risk prediction, but to compare the relative predictive behavior of simplified versus full ERAS frameworks. In this context, the fact that simplified pathways performed comparably for several outcomes suggests that key elements within ERAS may drive much of its beneficial effect, even when other components are not fully applied.

Full ERAS implementation remains the gold standard and is not challenged by our findings. However, achieving full compliance is difficult in many healthcare environments due to staffing limitations, infrastructural constraints, and challenges in multidisciplinary coordination. In such settings, validated simplified protocols may serve as realistic, impactful alternatives to the absence of any structured recovery program.

Our results support the concept of ERAS implementation as a continuum rather than an “all-or-nothing” strategy. Even with fewer components, simplified bundles retained non-negligible predictive capability for central postoperative outcomes. Thus, these abbreviated programs may serve as scalable, intermediate solutions, enabling hospitals to move toward broader ERAS adoption progressively. Over time, simplified protocols may act as practical entry points, facilitating institutional culture change, team engagement, and incremental infrastructure development necessary to achieve comprehensive ERAS compliance.

Although ERAS remains the gold standard, simplified protocols such as Nygren and RAPID performed comparably and, in some outcomes, even surpassed the predictive accuracy of the full protocol. Prioritizing individual components based on their effect on postoperative outcomes simplifies the guidelines. The present study contributes to this need by showing which simplified protocols align most closely with the predictive capacity of the full ERAS program. While this study was not designed to isolate the impact of single interventions, the comparative performance of the simplified protocols indirectly highlights clusters of elements that may be particularly influential. These insights, combined with existing evidence on core components, emphasize the need for future research to define a minimal yet effective ERAS “core set” tailored to different clinical environments. In this way, simplification would not aim to diminish ERAS principles but rather to support their broader and more consistent implementation.

The variability in adherence even within a structured ERAS program underscores the difficulty of achieving full compliance. The ability of simplified versions to produce similar predictive behavior for outcomes such as stoma-free recovery, complications, and readmissions suggests that a reduced set of high-impact measures may approximate the benefits of the full pathway, particularly in settings where universal implementation remains unrealistic.

The ERAS study program was contrived by the following interventions: preadmission counseling, preoperative optimization, preoperative nutritional support, management of anemia, no premedication, postoperative nausea and vomiting (PONV) prevention, antibiotic prophylaxis, selective bowel preparation, reduced fasting duration, standard anesthetic protocol (multimodal anesthesia and short-acting anesthetic agents), intraoperative fluid therapy, maintenance of normothermia, minimally invasive surgery, avoidance of prophylactic drains and nasogastric tube, minimal use of opioids, thromboprophylaxis, postoperative fluid therapy (avoidance of salt and water overload and avoidance of excessive postoperative weight gain), early removal of urinary catheter, stimulation of gut motility, postoperative nutritional care (nutritional support and early oral nutrition), and early mobilization.

There were four different simplified programs analyzed. The RAPID protocol encompasses only four interventions on patients submitted to elective laparoscopic and open colorectal resections, leading to a significantly lower length of stay (LOS) [12]. Avoidance of nasogastric tubes, of salt and water overload, and early removal of urinary catheter were grouped as the first item (remove). The other three were early mobilization (ambulate), minimal use of opioids (postoperative analgesia), and early oral nutrition (introduce diet). In the present study, the RAPID protocol demonstrated some utility for predicting all outcomes except readmissions. Thus, it is possible to implement a program with a smaller number of interventions with a somewhat positive effect on patient recovery.

Aarts et al. developed a multicenter prospective cohort of patients submitted to elective colorectal surgery. They found that compliance with the 11 program's interventions was associated with improved outcomes on both laparoscopic and open surgeries, with an impact significantly higher in the open group [13]. The interventions were as follows: preadmission counseling, no premedication, reduced fasting duration, selective bowel preparation, multimodal anesthesia, intraoperative fluid therapy, avoidance of intraoperative devices (nasogastric tubes and prophylactic drains), early mobilization, early feeding, stimulation of gut motility, and early removal of urinary catheter. A high adherence to the protocol led to a significantly greater impact in optimal recovery (discharge in five days without severe complications, no anastomotic leaks, no readmission, nor mortality in 30 days). In the present study, ERAS and Aarts were fairly adequate for predicting the absence of stoma, as well as overall and severe complications.

Basse et al. implemented a seven-item fast recovery program on patients undergoing open colonic resection. The interventions included no premedication, multimodal anesthesia, short-acting anesthetic agents, minimal use of opioids, early removal of urinary catheter, stimulation of gut motility, and a well-defined nursing care program (early mobilization/feeding). They found a significant reduction in LOS and complication rates compared to a control group (p <0.05) [14]. As for Nygren et al., the same was contrived for open elective colorectal surgeries with a 12-item program, apart from reducing complications, which was observed only in colonic resections [15]. These findings align with the laparoscopy and fast-track multimodal management (LAFA) trials, which state that optimal patient care is achieved by implementing a fast-track program [17]. The interventions contained in Nygren’s program were: preadmission counseling, antibiotic prophylaxis, selective bowel preparation, multimodal anesthesia, avoidance of prophylactic drains and nasogastric tube, minimal use of opioids, thromboprophylaxis, stimulation of gut motility, nutritional support, early oral nutrition, and early mobilization. Basse reasonably predicted mortality, and both programs were favorable for predicting the absence of general and severe complications and stoma.

However, readmission remains a widely debated topic in fast-track programs, as it may be a potential consequence of reducing the LOS [18]. Nygren et al. reported a significantly higher readmission rate in the fast-track group (15% vs. 4%, p<0.001). Similarly, Basse observed an increased readmission rate in the fast-track group (12% vs. 4%), though this difference was not statistically significant (p>0.05). Ahmed et al., in a systematic review, found that high compliance with a fast-track program is significantly associated with a shorter LOS but also with an increase in readmissions [10]. However, these findings are not consistent across all studies, as some have found no statistically significant difference in readmission rates between fast-track programs and regular care [19,20]. However, our study found that high adherence to the fast-track program applied by Nygren et al. was limited, but helpful for predicting readmissions.

Among the components used by Nygren et al., other studies have found that interventions such as immediate oral diet and epidural anesthesia have been shown to reduce postoperative morbidity as single interventions [21,22]. Several studies have confirmed the effect of epidural anesthesia in reducing postoperative ileus rates [23]. However, it is not clear whether the individual benefits of epidural anesthesia in comparison to early oral nutrition and mobilization. The study by Basse et al. shows that treatment with epidural analgesia may not solely lead to an improved recovery of gastrointestinal function unless combined with avoidance of routine use of nasogastric tubes, early oral nutrition, and early mobilization [14].

Nygren et al. noted that epidural anesthesia was routinely used even before implementing their fast-track program, suggesting that other protocol elements may have contributed to the improved outcomes observed in their study [15]. In opposition, balanced fluid therapy was not obtained, even though it has been shown to improve postoperative bowel function and reduce morbidity in other studies [23]. If the absence of optimized fluid balance influenced the analyzed outcomes, it is not known for sure, even though fluid excess may contribute to increased morbidity because of its cardiopulmonary and ileus risk [24]. Thus, even though there was a high adherence to an intervention such as epidural anesthesia in both groups of comparison (control and fast-track), as well as the complete absence of another intervention such as balanced fluid therapy, the outcomes were still favorable for the fast-track program.

In this sense, it could be said that these are nonessential items. The identification of core elements could be considered the solution for simplifying fast-track programs and facilitating implementation. Kehlet proposes that there are five key components for ERAS in colorectal surgery: preoperative patient information, thoracic epidural anesthesia in open (but not laparoscopic) surgery, avoidance of fluid overload and hypovolemia, no nasogastric tube, and combined with early oral feeding and mobilization [25]. So, both epidural anesthesia and balanced fluid therapy are contemplated as essential, even though their impact was minimal in Nygren’s study.

To this extent, Portilho et al. evaluated adherence to a 22-item ERAS program in elective colorectal surgery [6]. The authors found that the single interventions significantly associated with reducing severe complications were avoiding prophylactic drains, minimal use of opioids, avoidance of salt and water overload, postoperative nutritional support, and early mobilization. A meta-analysis evaluated 53 studies with 56 different groupings of fast-track components, of which only two used the same combination. The adherence was higher than 80% for five core items: preoperative counseling, early mobilization, early oral feeding, epidural analgesia, and no nasogastric intubation [26]. Balanced fluid therapy and epidural anesthesia were identified as essential in the first and latter studies, respectively.

In addition, in Gianotti et al.’s meta-analysis, it was found that when comparing studies containing at least four core components with those lacking said items, the positive effects of fast-track programs on morbidity and LOS were unaffected in comparison to conventional care [relative risk (RR)=0.80; 95 % confidence interval (CI) 0.69-0.93 and RR=0.73; 95 % CI 0.61- 0.87, respectively]. In this way, the advantages of a fast-track program may reach a steady state after applying some of its elements. Thus, even though epidural anesthesia and balanced fluid therapy can be considered essential, it may also be possible to obtain the benefits of a fast-track program with the implementation of other non-core interventions.

Furthermore, the implementation and effect of an intervention can be influenced by the patient’s intrinsic risk. For example, nutritional support was evaluated by two different interventions in the ERAS study. Preoperative nutritional care with immunonutrition supplementation was indicated only for malnourished patients, and postoperative nutritional care was for everyone [6]. The preoperative supplementation for malnourished patients was not associated with a lower rate of complications, as the postoperative supplementation for everyone was. It could be inferred that the nutritional state was decisive for better outcomes.

Immunonutrition is still a widely discussed topic in the fast-track program context. Some studies have shown the benefits of perioperative supplementation in well-nourished and malnourished patients [27]. However, postoperative immunonutrition may increase morbidity in critically ill patients and is often recommended only for malnourished patients [28]. In this sense, some interventions may be potentiated when applied to patients with other singular characteristics, such as intrinsic risk or even other interventions used previously. Thus, in order to achieve effective simplified versions of fast-track programs, some interventions may be directed at specific patients.

The meta-analysis by Gianotti et al. suggests that the lack of additional improvement after the application of a number of core components may depend on the patient's own intrinsic risk [26]. Kehlet agrees that when outcomes are suboptimal, there is less need for adherence to the other, less relevant components in most protocols [25]. In such a manner, one recent modality of ERAS programs is the so-called same-day discharge protocols, or “hyper-ERAS”. They encompass a series of ERAS components for physiologically fit patients aiming for discharge on the day of surgery and have been shown to be feasible, with low risk of readmissions and comparable risks of morbidity [29].

In this way, simplifying programs is a challenge. There are a multitude of variables that need to be taken into consideration to improve outcomes. Some interventions interact with one another, the patient’s own risk, core items that must be prioritized, and several other components that can bring benefit even without the implementation of those considered essential. However, simplification is still possible. The positive effect of fast-track programs in morbidity over conventional care was observed regardless of the number (<10 vs. ≥10) of interventions used (RR=0.80; 95 % CI 0.66-0.98 and RR=0.75; 95 % CI 0.65-0.87, respectively) in Gianottis et al.’s meta-analysis.

Nevertheless, there is an overall lack of standardization when comparing each program’s items. For example, in the ERAS study, the avoidance of fluid overload and hypovolemia was divided into three items: intraoperative fluid management, avoidance of salt and water overload, and avoidance of excessive weight gain. The use of goal-directed fluid therapy could be an attempt to set a parameter, but it's not routinely implemented due to its high complexity, and even the 2018 ERAS Guidelines recommend it only for high-risk patients [30].

Even though some components are measured and compared in different ways, there is still vast evidence of their effectiveness as a whole. Gianotti et al. propose that the simple implementation of a standardized protocol appears sufficient to enhance patient care and morbidity outcomes, regardless of the number, combination, type, or strength of evidence associated with individual ERAS elements [26]. Nevertheless, its application as routine care remains challenging and slowly progressing [7]. Thus, simplified and standardized programs can facilitate implementation and enable accurate data collection to solve age-old problems such as predicting the occurrence of hospital readmission or determining which interventions have the most impact on postoperative outcomes.

The present study is a retrospective analysis with inherent limitations. Differences in the original structure of the protocols must also be acknowledged. The Basse and Nygren bundles were developed and validated in cohorts composed almost entirely of open colorectal operations. In contrast, our contemporary population reflects current standards of care, with 96.3% of procedures being minimally invasive. If analyzed strictly from a historical perspective, these discrepancies could suggest limited comparability. However, protocols are designed to be transportable beyond the context in which they were created, and advances in minimally invasive surgery (MIS) techniques and anesthetic care have reshaped postoperative risk profiles. Therefore, external validation in modern MIS-dominant cohorts is not only appropriate but scientifically necessary to establish whether simplified care bundles remain meaningful today. Rather than undermining contemporary applicability, the fact that these protocols were developed 10-20 years ago highlights the need to re-evaluate their performance under current surgical standards. Our findings help clarify how simplified ERAS-derived pathways function in the current MIS-dominant era, contributing evidence that may support the ongoing refinement and modernization of ERAS-based bundles.

## Conclusions

Simplified ERAS protocols, such as Nygren and RAPID, demonstrated competitive or even superior predictive performance in certain outcomes, such as stoma-free and avoiding postoperative mortality. While the ERAS protocol remains a robust standard, simplified versions may offer practical alternatives in specific scenarios.
